# Does the match between individual and group behavior matter in shoaling sticklebacks?

**DOI:** 10.1002/ece3.8581

**Published:** 2022-02-14

**Authors:** Sin‐Yeon Kim, Náyade Álvarez‐Quintero, Neil B. Metcalfe

**Affiliations:** ^1^ 16784 Grupo Ecoloxía Animal Torre CACTI Centro de Investigación Mariña Universidade de Vigo Vigo Spain; ^2^ 3526 Institute of Biodiversity, Animal Health and Comparative Medicine University of Glasgow Glasgow UK

**Keywords:** collective behavior, *Gasterosteus aculeatus*, group living, growth, metabolic rate, sociability

## Abstract

In animals living in groups, the social environment is fundamental to shaping the behaviors and life histories of an individual. A mismatch between individual and group behavior patterns may have disadvantages if the individual is incapable of flexibly changing its state in response to the social environment that influences its energy gain and expenditure. We used different social groups of juvenile three‐spined sticklebacks (*Gasterosteus aculeatus*) with experimentally manipulated compositions of individual sociability to study the feedback between individual and group behaviors and to test how the social environment shapes behavior, metabolic rate, and growth. Experimentally created unsociable groups, containing a high proportion of less sociable fish, showed bolder collective behaviors during feeding than did corresponding sociable groups. Fish within groups where the majority of members had a level of sociability similar to their own gained more mass than did those within mismatched groups. Less sociable individuals within sociable groups tended to have a relatively low mass but a high standard metabolic rate. A mismatch between the sociability of an individual and that of the majority of the group in which it is living confers a growth disadvantage probably due to the expression of nonadaptive behaviors that increase energetic costs.

## Graphical abstract

In animals living in groups, individual differences in social behavior have substantial consequences for social structure and functioning, thereby driving collective behaviors. However, a mismatch between individual and group behavior patterns may incur fitness costs if the individual is incapable of changing its state or behavior in response to the social environment. By experimentally grouping sociable and unsociable stickleback fish, we show that fish grew better when belonging to a group in which the sociability of the majority of members matches their own.

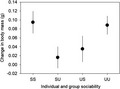

## 1 INTRODUCTION

It is now well known across diverse animal taxa that individuals differ consistently from each other in their behavior patterns (Bell et al., 2009; Réale et al., 2010) and that these differences often carry fitness consequences at the individual level (Moiron et al., 2020; Smith & Blumstein, 2008). Individuals adjust their behaviors according to their current condition and internal state, producing consistent individual differences in behavior patterns (Dingemanse & Wolf, 2010; but see Niemelä & Dingemanse, 2018). In animals living in groups, the existence of individual differences in behavior patterns is also expected to have substantial consequences for social functioning and social structure within the groups (Hui & Pinter‐Wollman, 2014; Modlmeier et al., 2014; Webster & Ward, 2011; Wolf & Krause, 2014). Differences in social behavior, in particular, may play a fundamental role in driving the collective behaviors of animal groups, determining how they move, forage, and avoid predators. For example, a recent study on a fish species showed that individual's propensity to stay near others (“sociability”) predicts differences in structure, cohesion, and movement dynamics between groups (Jolles et al., 2017). Thus, individual sociability may influence the levels of group activity and foraging behavior (McDonald et al., 2016), which in turn affects the individual's energy turnover (acquisition and expenditure). However, few studies have investigated how the social environment within a group influences the performance of individuals that differ from each other in their behaviors and states (but see Cote et al., 2011).

In order to study how the social environment influences individual behavior, life history, and physiology, it is perhaps necessary to understand the feedback between individual sociability and collective group behaviors (Aplin et al., 2013; Jolles et al., 2017). A high level of average sociability of group members does not necessarily mean that they would form a more dynamic and mobile group than less sociable individuals. A recent study showed that, in a fish species, groups with a high average individual sociability moved relatively slowly and with little alignment, although sociable individuals tended to form a relatively cohesive group by staying closer to each other (Jolles et al., 2017). This is probably because sociability is often related to other behaviors; for example, unsociable individuals are proactive and tend to lead their groups to move dynamically, whereas sociable individuals are reactive and conform to other group members' behavior (Bergmüller & Taborsky, 2010; Bevan et al., 2018; McDonald et al., 2016).

Many of the collective behaviors in groups are known to have relatively strong consequences for energy gain and expenditure (Mathot et al., 2019). For instance, the collective decision‐making and foraging behaviors of a group are closely associated with its access to resource and intake rate; the group's sustained speed, maximum sprint speed, and total distance of movement influence its energy expenditure (Hansen et al., 2020; McLean et al., 2018). Since, at the individual level, the energetic benefits and costs are determined through the interaction between individual behavioral processes and collective group performances (Couzin & Krause, 2003; Jolles et al., 2020), the energetic efficiency of collective behavior may differ among individuals within the same group. Thus, the match and compatibility between individual and group behaviors may have consequences for individual fitness through their effects on condition, growth, and survival. For example, a sociable and reactive individual that has a low energy turnover rate (Réale et al., 2010) may express a relatively proactive lifestyle when it belongs to a group that moves consistently fast (Jolles et al., 2018). This mismatch may incur fitness costs if the individual is incapable of also adjusting its physiology in response to the social environment that it experiences.

One of the key physiological traits related to behavior is an individual's metabolic rate (Glazier, 2015; Mathot et al., 2019). Its minimal rate of energy metabolism (basal or standard metabolic rate for endo‐ and ectotherms, respectively) is important since this reflects the minimal energetic cost of living and is one of the primary traits underlying organismal performance (Auer et al., 2015); it has also been shown to evolve in parallel with different behavioral and life‐history traits (Auer et al., 2018). Minimal rates of metabolism are generally constant under stable conditions, and it is commonly believed that metabolic rate drives the rates of behavioral and life‐history processes (Galliard et al., 2013). For example, a high metabolic rate can promote higher activity, exploration, and a more productive lifestyle (Biro & Stamps, 2010; Careau et al., 2008). However, there is increasing evidence that metabolic rate also responds to the rates of other biological processes, such as growth and food intake, which change according to the natural and social environment (Auer et al., 2015; Glazier, 2015; Norin et al., 2016; Norin & Metcalfe, 2019). The degree of metabolic flexibility varies among individuals (Auer et al., 2015), and an individual's metabolic flexibility may represent its capacity to change its behavior and life history in response to its social environment.

Here, we studied whether and how the social environment shapes the behavior, metabolic rate, and life histories of individuals that differ in sociability, by using an experiment on the three‐spined stickleback (*Gasterosteus aculeatus*). In this species, the social environment is particularly important during early life because juveniles tend to form groups and have intense social interactions (Östlund‐Nilsson et al., 2007). We have shown in our recent studies that the propensity of juvenile sticklebacks to stay near others is highly repeatable within individuals (when measured repeatedly within a short time period in a constant environment), heritable and genetically integrated with other functional traits (Kim & Velando, 2015), yet flexibly changes in response to predation risk (Kim, 2016). We created experimental social groups by manipulating the proportions of individual behavior types (i.e., whether the group was made up of relatively sociable or unsociable individuals) to examine whether juvenile sticklebacks change their social behavior in response to their social environment (sociable vs. unsociable environment). Since in this species, social propensity has a strong effect on collective group behavior (Harcourt et al., 2009; Jolles et al., 2017), we also examined how the sociability of the experimental groups influences their feeding behavior. Finally, we examined whether this interaction between individual sociability and social environment influences physical condition (i.e., critical swimming speed) and metabolic rate of the fish and has consequences for their growth and survival.

## 2 MATERIAL AND METHODS

### 2.1 Study population

A total of 140 juvenile three‐spined sticklebacks (aged approximately 3–5 months) were captured with hand nets in the Rio Sar (Galicia, Spain) in July 2019 and then housed individually in 8‐L tanks in an indoor facility. The tanks were connected to closed flow‐through water systems (30 tanks per system) in which water was continuously filtered, aerated, and temperature‐controlled. The lateral walls of the tanks were opaque, so preventing visual contact between individual fish. The natural seasonal photoperiod and water temperature in the region (light:dark 15 h:9 h and 20°C in July) were simulated in the holding tanks by programmed illumination and flow‐through water cooling system. Fish were fed daily on a commercial pelleted diet (Gemma Micro, Skretting, Norway). Any mortality in the fish was recorded daily.

### 2.2 Sociability assay and experimental groups

Before testing their initial level of individual sociability, focal fish were acclimatized in the individual tanks for 6 or 7 days to avoid direct social interactions with conspecifics, which might affect their attraction to a group of conspecifics. A total of 130 fish survived this acclimatization process. The sociability of each fish was then tested in a Perspex observation tank (35 × 15 × 15 cm) that was partitioned into two compartments, termed the focal fish zone and the stimulus fish zone (25 cm and 10 cm length), by means of a transparent barrier. The observation tank was filled with water from the holding aquaria system to reduce the stress to both focal and stimulus fish and allow olfactory stimulation. The stimulus fish zone contained three juvenile sticklebacks. A focal fish was netted carefully from its home tank, transferred to the focal fish zone of the observation tank, and released at the opposite end from the transparent barrier. The behavior of the focal fish was then recorded from above without disturbance for 180 s by using a digital camera mounted on a tripod. Because the behavior was assessed immediately after the focal fish was released in the observation tank without acclimation, the observed behavior could be influenced by handling stress to some extent, but this would be standardized since the procedure was the same for all fish. After this behavior assay, the focal fish was weighed to 0.001 g by using a digital balance, measured to the nearest 0.5 mm (standard length), and permanently marked with color elastomer tags (Northwest Marine Technologies, Shaw Island, WA, USA) for individual identification under a low dose of benzocaine anesthetic before being returned to its home tank. Each focal fish was used as stimulus for three consecutive sociability assays of other individuals after first itself being tested for sociability. The social behavior of all fish, except the first three stimulus fish, was assayed and recorded during two consecutive days (*N* = 127). There was no consistent difference in size (i.e., standard length) of the focal fish and that of the average size of the three stimulus fish (paired *t* test: *t*_126_ = 0.044, *p* = .965; mean length ± SE: 28.2 ± 0.2 mm, *N* = 127). The level of sociability (see below) of the focal fish was not correlated with the average level of sociability of the stimulus fish (*r* = 0.017, *N* = 127, *p* = .850).

Videos were analyzed using the automated behavior tracking software ToxTrac v2.84 (Rodriguez et al., 2018) to measure the propensity of the focal fish to stay near others. In each trial, we measured the total time that the focal fish spent in the socialization area, that is, 4 cm wide section of the focal fish zone adjacent to the stimulus fish zone, during the first 120 s after the focal fish was released from the net. We analyzed only the first 120 s to improve data distribution because most fish (96%) approached the barrier within this time then spent most time in the socialization area. In previous studies, we used the time taken for a fish to reach the barrier between the focal fish and stimulus fish zones as a measure of sociability (Kim, 2016; Kim & Velando, 2015). However, we think that the time spent in the socialization area is a slightly better index of individual sociability than the time taken to approach the barrier, because some fish rapidly approached the barrier as soon as they were released in the focal fish zone but then spent relatively little time socializing with the stimulus group, although the two measurements of social propensity were strongly correlated with each other in this study (linear regression: *r* = .874, *F*_1,125_ = 403.93, *p* < .001).

After the sociability assay, the focal fish were individually housed in their home tanks until we created ten experimental groups of nine individuals based on their individual sociability, that is, the time spent in the socialization area, in early August (for a similar approach, see Cote et al., 2012). A total of 45 fish with the highest sociability scores that spent more time socializing with the stimulus group were classified as “sociable” individuals (mean ± SE time: 111.4 ± 0.7 s), and the 45 fish with the lowest scores as “unsociable” individuals (64.1 ± 4.6 s). The rest of the fish with intermediate sociability scores were excluded from the remainder of the experiment. The relatively sociable and unsociable fish were systematically allocated according to their sociability scores and elastomer marks (to allow individual identification within the group) into five “sociable” groups, each made up of six relatively sociable individuals and three relatively unsociable individuals, and five “unsociable” groups, composed of three sociable and six unsociable individuals. Thus, this experimental grouping created four different types of fish, relatively sociable fish within sociable groups (SS, *N* = 30), relatively unsociable fish within sociable groups (US, *N* = 15), sociable fish within unsociable groups (SU, *N* = 15), and unsociable fish within unsociable groups (UU, *N* = 30). Since the number of fish between these four categories was unbalanced, subsequent statistical analyses of growth rate and changes in sociability are based on the 15 most sociable fish and all 15 unsociable fish within the sociable groups, and the 15 most unsociable fish and all 15 sociable fish within the unsociable groups. However, the results did not change when all experimental fish were included in the analyses.

The experimental fish groups were housed in 10 outdoor PVC tanks (71 × 111 × 39 cm, filled with 260 L water), which were large enough to allow the fish to either shoal or be dispersed. Each tank contained a ceramic hollow brick and a roof tile under which fish could shelter, and water was continuously filtered and aerated (Figure S1).

### 2.3 Observation of group feeding behavior and individual sociability after experimental grouping

The fish in the experimental group tanks were fed daily to satiation on the same commercial food as above. Food was always provided at the same position, the opposite end from the water filter (Figure S1) and at the same hour of the day, 12 noon. The food pellets initially floated but eventually sank to the bottom if uneaten; any uneaten food on the bottom of the tanks was removed every day 20 min before the next feeding event. When the experimental groups were first created, most fish did not emerge from the shelter or actively feed when food was provided, but the fish became increasingly fast to respond to food over time. In order to examine how the composition of groups in terms of individual sociability influenced their feeding behavior, we observed the fish groups once every week for six weeks (in August and September), starting from 11 days after the onset of group living.

In each observation, the observer stood quietly and unobtrusively at the feeding point of an experimental tank and first counted the number of fish present outside the shelter. Then, she provided pelleted food in the usual location, remained at the same observation position, and counted the number of fish feeding on the surface of water at 1‐min intervals for 5 min. After the 5‐min observation, the observer again counted the number of fish present outside the shelter. Although some individuals were identifiable by their elastomer tags during the observation, we did not record fish identity because the detection of individual fish would be biased due to different visibility of different color tags. Many fish emerged from their shelter after food was provided, but some individuals stayed close to the bottom of the tank and during the observation period were only seen to feed on the few pellets that sank whereas the others actively fed on the majority of pellets that remained floating on the surface of water. For statistical analyses, we used the maximum number of fish feeding on the surface of water during the 5‐min observation in each trial, which represents the group's willingness to actively feed in the presence of the observer, and so is a measure of its general boldness. The maximum number was strongly correlated with the average number of fish feeding on the surface of water during the observation (*r* = .962, *N* = 60, *p* < .001).

After two months of living in the experimental group (i.e., in early October), individual sociability was assessed again in the same way as before, but this time the experimental fish were not used as the stimulus fish. Those fish from the original population that initially had intermediate sociability scores, and so were excluded from the experimental groups, were used as the stimulus fish in up to six sociability assays. Fish were measured and weighed before being returned to their experimental group tanks.

### 2.4 Metabolic rates and swimming performance

After the second sociability assay, swimming performance and metabolic rates were determined in a subsample of randomly selected focal fish (SS, *N* = 5; US, *N* = 4; SU, *N* = 4; UU, *N* = 5) during 18 consecutive days (one individual per day) by using an intermittent‐flow swim tunnel respirometer system (Loligo Systems, Viborg, Denmark). This respirometer system consisted of a 170 ml swim tunnel, a 20‐L buffer tank, a flush pump, a variable‐voltage motor, a fiber optic oxygen sensor, a temperature sensor, a data acquisition instrument, and an automated system controller instrument. The oxygen sensor and flow‐controlling motor were calibrated following the manufacturer's instructions prior to use. The tube‐shaped swim tunnel respirometer was submerged in the buffer tank in which water temperature was maintained at 15.4 ± 1°C (the same as the average water temperature in the group holding tanks). Dissolved oxygen inside the respirometer was registered every second and water was automatically flushed into the respirometer every 5 min following a programmed protocol (90 s flush, 30 s waiting and 180 s measurement) by using the manufacturer's AutoResp software (Loligo Systems, Viborg, Denmark). During each session of measuring metabolic rate and swimming performance, the room was kept dim during the day (11 h) or completely dark at night (13 h) to prevent disturbance to the test individual. To mitigate the energetic costs of digestion, which lead to an elevation in metabolic rate, fish were not fed for 24 h prior to the measurements.

We simultaneously measured metabolic rate and swimming performance by following the critical swimming speed (*U*_crit_) protocol combined with intermittent‐flow respirometry (reviewed in Norin & Clark, 2016). Before each session, a focal fish was transferred from its outdoor tank to the laboratory (10 m distance) and acclimated in an individual tank for 2 h. Each session started at 12 noon by introducing the fish into the swim tunnel respirometry chamber. It was left undisturbed in the chamber at a minimum flow speed for mixing of the water within the system without enforcing swimming activity for the first 22 h to measure its standard metabolic rate (SMR) and then subjected to the swimming performance test inside the same chamber. During this test, the water velocity was first set to 1.5 cm s^−1^ (i.e., approximately 0.5 body length s^−1^) by using a motor‐driven propeller. The fish was acclimated at the low flow rate for 20 min, and then, flow rates were increased by a further 1.5 cm s^−1^ every 5 min until exhaustion, when it could no longer keep its position in the tunnel. The time and water velocity when the fish became fatigued was recorded. After the exhaustive exercise, the fish remained in the respirometer at a minimum current speed for 30 min to measure maximal metabolic rate (MMR) during recovery. During the whole session (SMR measurement, swimming performance, and MMR measurement) of up to 24 h, changes in dissolved oxygen inside the respirometer were measured every second as mg O_2_ L^−1^. Oxygen levels were always maintained above 84% during measurements.

Metabolic rate at each interval was calculated in mg O_2_ h^−1^ as follows:

$${\mathrm{MO}}_{2}=\;\frac{\Delta O_{2}}{\Delta t}\left(V_{r}‐V_{f}\right)$$

where ΔO_2_/Δ*t* is the change in dissolved oxygen over time (mg O_2_ L^−1^ h^−1^), *V_r_* and *V_f_* are the volumes (L) of the respirometer and the fish. The fish volume is assumed equivalent to the mass with a density of 1 kg L^−1^. Individual SMR was estimated as the average of the lowest 10th percentile of all measurements during the first 22 h. MMR was determined as the highest rate of MO_2_ achieved within 30 min after the swimming performance test. Individual aerobic scope (AS) was calculated as MMR minus SMR.

*U*_crit_, a standard measure of prolonged swimming performance during which a fish is forced to swim against an incrementing water flow until fatigue (Brett, 1964; Kolok, 1999), was determined as follows:

$$U_{\mathrm{crit}}=U_{f}+U_{s}\frac{t_{f}}{t_{s}}$$

where *U_f_* is the highest speed (cm s^−1^) maintained for an entire interval (*t_s_* = 5 min), *U_s_* is the speed increment between intervals (1.5 cm s^−1^) and *t_f_* is the time until fatigue in the final speed interval.

### 2.5 Statistical analyses

We tested whether group behaviors during feeding changed across repeated trials over 6 weeks and differed between sociable and unsociable groups by using generalized linear mixed models (GLMMs) with a binomial error distribution and logit link function. Since the total number of fish in a group varied due to mortality, the proportions of fish outside the shelter before feeding and after feeding and the proportion of fish feeding on the surface of the water in each observation were used as response variables in the analyses. Experimental treatment (sociable or unsociable group), trial (weeks 1–6), and their interaction were included as fixed effects, and trial and group identity were included as nested random effects.

Within‐individual repeatability of social behavior measured before and after the experimental group‐living period (2 months) was assessed using the *rpt* function of the rptR package in R 3.6.1 (Stoffel et al., 2017). Our model included time as a fixed effect and individual fish as the grouping factor. In addition, individual sociability measured during the treatment (i.e., two months after the onset of treatment) was analyzed in a linear mixed effect model (LMM), including treatment (sociable or unsociable group), initial sociability category (sociable or unsociable individual), and their interaction as fixed effects and group identity as a random effect. For this analysis of individual sociability, arcsine‐transformed proportion of time spent in the socialization area was used. Changes in body mass and standard length during the treatment were analyzed in LMMs, including treatment, initial sociability category, treatment × initial sociability category, and initial measurement (mass or length) as fixed effects and group identity as a random effect. Changes in body mass and length were calculated as differences in the measurements between before and after the two‐month experimental group‐living treatment. The survival of fish during two months of the experimental group‐living period was analyzed by using a Cox proportional hazard model, including treatment, initial sociability category, and their interaction as fixed effects and group identity as a random effect.

Swimming performance (*U*_crit_) and metabolic rate measurements (SMR, MMR, and AS) were first analyzed to test the effect of experimental treatment by using LMMs, including treatment, individual sociability category, body mass (in the analyses of SMR, MMR, and AS) or length (in the analysis of *U*_crit_), and their two‐ and three‐way interactions as fixed effects and group identity as a random effect. Then, we explored the relationships between current individual sociability measured in October and *U*_crit_, SMR, MMR, and AS after the experimental treatment in LMM analyses, including time spent in the socialization area (continuous variable), body mass or length, and their two‐way interaction as fixed effects. The effects of treatment and post‐treatment individual sociability on swimming performance and metabolic rate measurements were analyzed in separate models to avoid overparameterization due to the small sample size (*N* = 18).

GLMM and LMM analyses were performed by using the *glmer* and *lmer* functions of the LME4 package in R 3.6.1 (Bates et al., 2015). In GLMM analyses, significance of a fixed term was assessed by a likelihood ratio test (LRT) comparing models with and without the given term. In LMM analyses, significance was assessed using a type III ANOVA test, where degrees of freedom were calculated by Satterthwaite's approximation, using the lmerTest package (Kuznetsova et al., 2017).

## 3 RESULTS

### 3.1 Initial categorization of fish sociability

There was no significant difference in the initial body mass of individuals categorized as relatively unsociable and sociable in July, before the experimental treatment groups were formed (means ± SE, sociable fish: 0.261 ± 0.010 g; unsociable fish: 0.285 ± 0.011 g; *t*_88_ = 1.571, *p* = .120), nor in their standard length (sociable: 27.8 ± 0.3 mm; unsociable: 28.6 ± 0.3 mm; *t*_88_ = 1.746, *p* = .084).

### 3.2 Social group effects on behaviors

**FIGURE 1:**
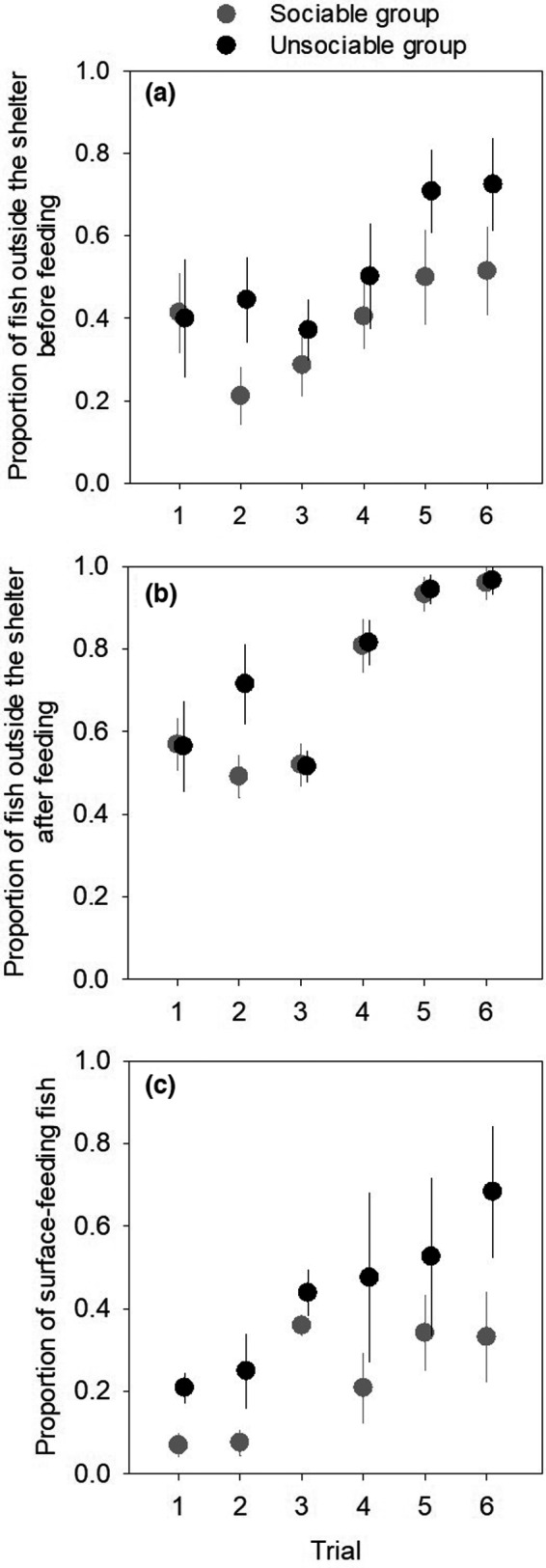
Collective behaviors of experimental groups during feeding. Proportions of fish (a) seen outside the shelter before feeding, (b) seen outside the shelter after feeding, and (c) feeding on the surface of water in weekly observations during the period of experimental group‐living (mean ± SE)

Collective feeding behaviors differed between the sociable and unsociable groups and changed over time throughout the experimental group‐living period. The proportion of fish found outside the shelter before each feeding event was significantly higher in the unsociable groups than the sociable groups and increased throughout the six week period of observations in both treatment groups (Figure 1a; GLMM: treatment: $\chi_{1}^{2}$ = 6.079, *p* = .014; trial: $\chi_{1}^{2}$ = 7.075, *p* = .008; treatment × trial: $\chi_{1}^{2}$ = 0.943, *p* = .332; see also Table S1). The proportion of fish outside the shelter after the feeding event, which include those that emerged from the shelter when food was provided, also increased over time, but did not differ between the sociable and unsociable groups (Figure 1b; GLMM: treatment: $\chi_{1}^{2}$ = 1.383, *p* = .240; trial: $\chi_{1}^{2}$ = 9.942, *p* = .002; treatment × trial: $\chi_{1}^{2}$ = 0.063, *p* = .802; Table S1). In the last observation (the sixth week), almost all fish in the group (on average 96%) were outside the shelter by the end of the 5 min feeding period in both treatments. Although similar numbers of fish were outside the shelter during the feeding events in the sociable and unsociable groups (Figure 1b), the proportion of fish actively feeding on the surface of water was significantly higher in the unsociable groups while increasing over time at similar rates in both group types (Figure 1c; GLMM: treatment: $\chi_{1}^{2}$ = 11.875, *p* < .001; trial: $\chi_{1}^{2}$ = 10.656, *p* = .001; treatment × trial: $\chi_{1}^{2}$ = 0.222, *p* = .638; Table S1).

**FIGURE 2:**
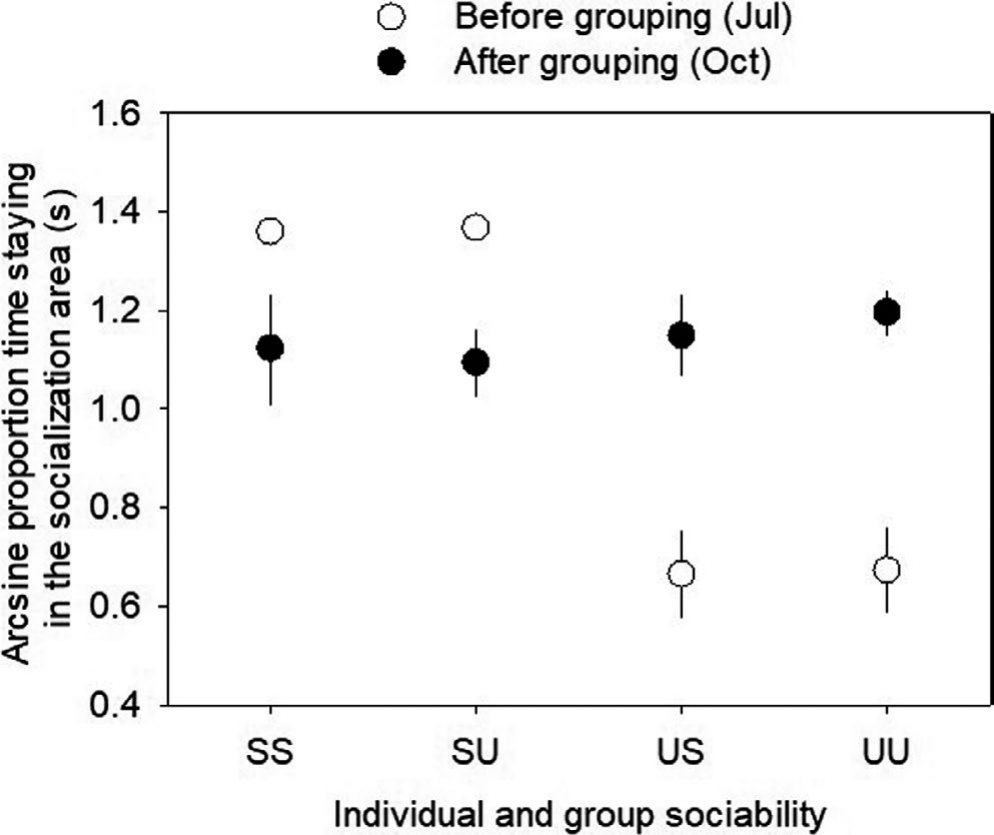
Change in individual sociability of juvenile sticklebacks. Arcsine‐transformed proportion of time fish spent in the socialization area during the sociability assays before and after the experimental group‐living according to the initial individual sociability category and treatment group (SS: sociable individuals within sociable groups; SU: sociable individuals within unsociable groups; US: unsociable individuals within sociable groups; UU: unsociable individuals within unsociable groups; mean ± SE (some SE values being too small to see); before, *N* = 60 fish; after, *N* = 36 fish)

After two months living in the experimental social groups, individual propensity to stay close to others became similar between initially sociable and unsociable fish within both sociable and unsociable groups (Figure 2). Thus, arcsine‐transformed sociability measured in October was influenced by neither the experimental treatment (LME: *F*_1, 8.39_ = 0.007, *p* = .937), the initial sociability category (*F*_1, 48.18_ = 0.074, *p* = .787) nor their interaction (*F*_1, 47.19_ = 0.087, *p* = .769). Repeatability analysis showed that the levels of sociability (time spent in the socialization area) measured before and after the experimental group‐living were not repeatable within individuals (*R* = 0, 95% CI: 0–0.181, *p* = 1).

### 3.3 Social group effects on growth and survival

**FIGURE 3:**
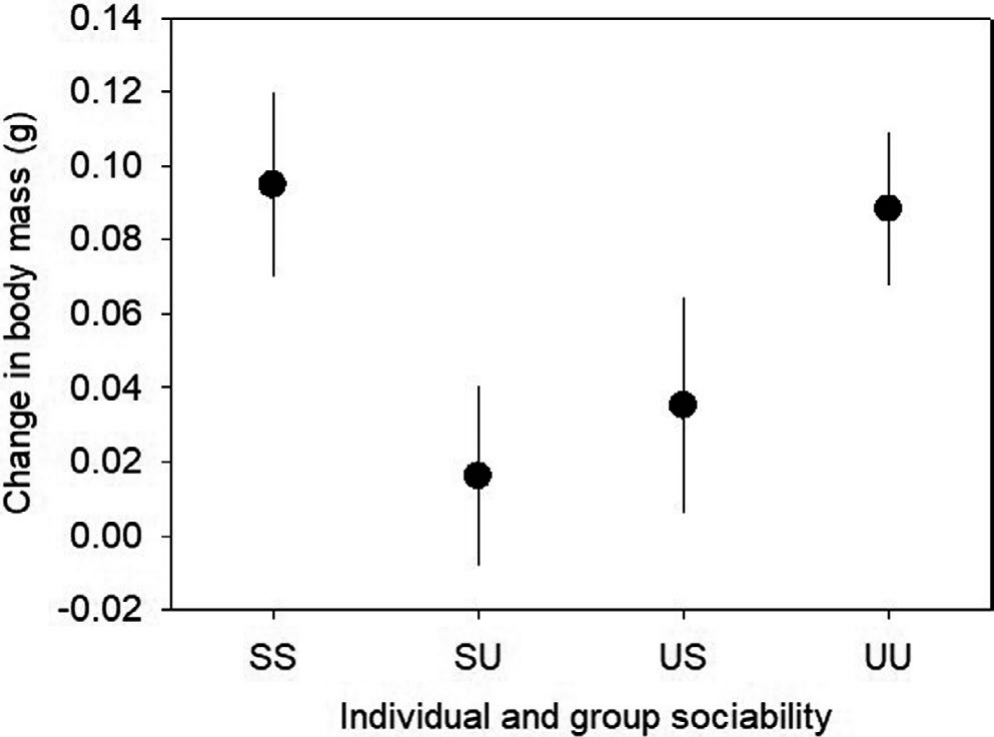
Body mass gain during the two‐month period of experimental group‐living in relation to the initial individual sociability category and treatment group (mean ± SE)

The LMM analysis of change in individual body mass during the experimental group‐living period, calculated as mass in October minus mass in July, showed a statistically significant interaction effect of treatment and initial individual sociability (*F*_1, 31_ = 7.875, *p* = .009; Table S2). Fish initially categorized as sociable individuals gained more mass during the group‐living period when they grew within the sociable groups than within the unsociable groups, whereas unsociable fish grew relatively better within the unsociable groups (Figure 3). However, there was no effect of the treatment, initial individual sociability, initial length, and treatment × initial sociability on change in standard length during the group‐living period (Table S2).

Among the 60 focal fish (15 SS, 15 SU, 15UU, and 15 US), 11 SS, 9 SU, 10 UU, and 6 US individuals survived the two‐month period of experimental grouping. Although unsociable fish allocated to the sociable groups showed a tendency to survive less than those in other social settings, a Cox proportional hazard model, including group identity as a random effect, showed no effect of treatment, initial individual sociability, or their interaction on survival (*p* > .999). Under the same husbandry conditions, sticklebacks grown in our laboratory normally show extremely low mortality. The fish used in this experiment showed higher mortality than expected perhaps because the transition from acclimatization in isolation to group‐living conditions within only three weeks after capture in the wild was stressful for these young fish. It is also possible that the density of the fish (9 individuals per 260 L) was below the optimal level in these large tanks, which are normally used in our laboratory to house approximately 30 juvenile or adult fish.

### 3.4 Swim performance and metabolic rates

**FIGURE 4:**
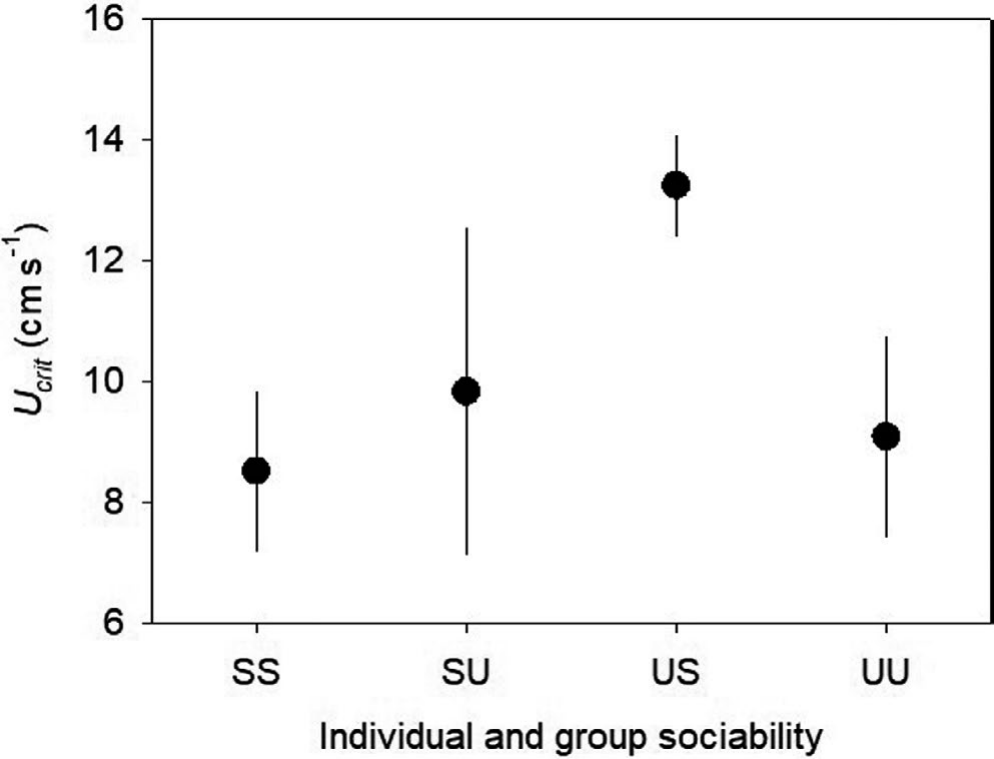
Critical swimming speed (*U*_crit_) measured after two months of the experimental group‐living, in relation to the initial individual sociability category and treatment group (mean ± SE)

**FIGURE 5:**
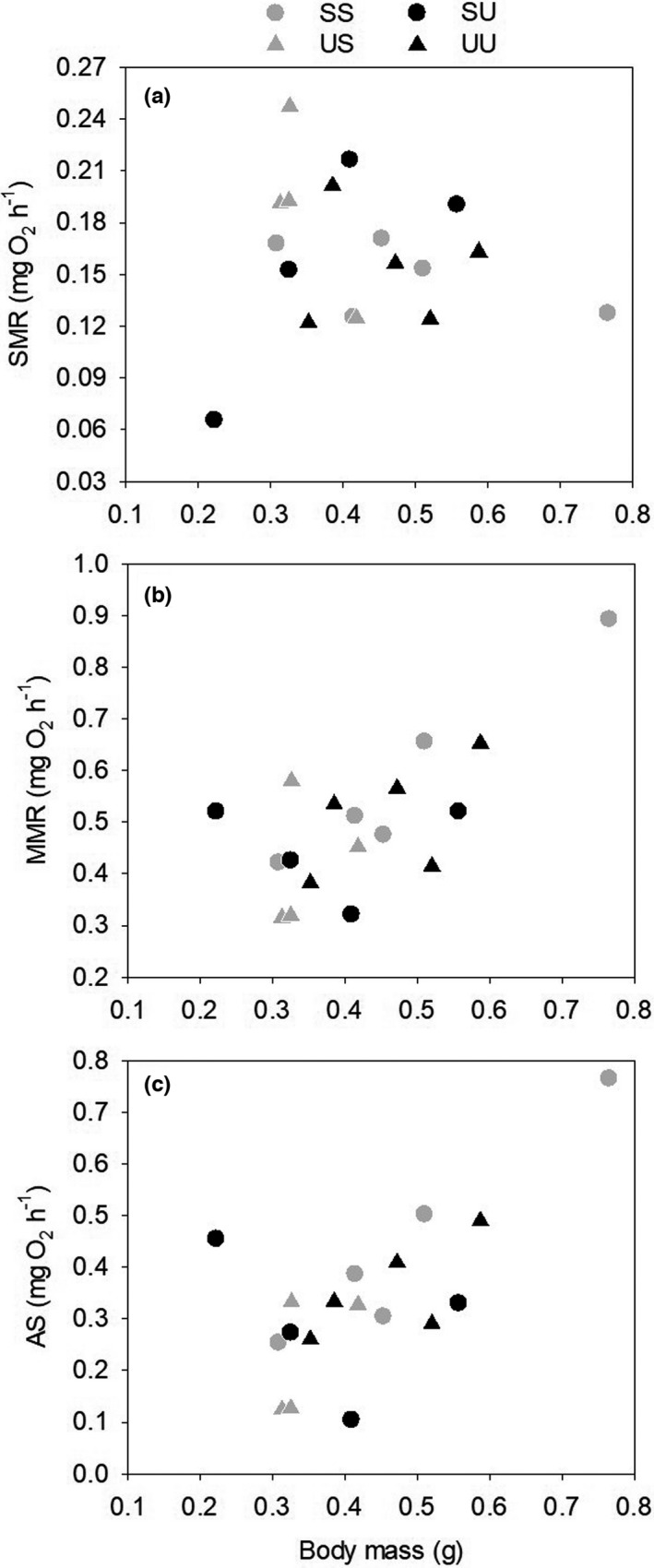
Relationships between body mass and (a) standard metabolic rate (SMR), (b) maximum metabolic rate (MMR), and (c) aerobic scope (AS) measured after the experimental group‐living, shown in relation to the initial individual sociability category and treatment group

The LMM analysis of *U*_crit_, testing the effects of treatment, initial individual sociability category, standard length, and their interactions, showed a statistically significant effect of treatment × initial sociability (*F*_1, 7.08_ = 6.081, *p* = 0.043; Table S3). Relatively unsociable individuals within the sociable groups (US) sustained a faster swimming speed (i.e., had a higher *U*_crit_) than the others (Figure 4). Similar analyses were performed for metabolic rates by including body mass (instead of length) as a covariate. There were statistically significant interacting effects of treatment × mass and initial sociability × mass on SMR (*F*_1, 9.3_ = 10.697, *p* = .009; *F*_1,11.7_ = 7.947, *p* = .016; Table S3). There was a positive trend linking mass and SMR in individuals within the unsociable groups (Pearson correlation: *r* = .476, *p* = .195), and a negative one within the sociable groups (*r* = −.576, *p* = .105), but neither relationship was significant (Figure 5a). Fish initially categorized as unsociable individuals and sociable individuals also showed not significant correlations between mass and SMR (unsociable: *r* = −0.481, *p* = .190; sociable: *r* = .221, *p* = .567). MMR was positively related to body mass (*F*_1, 14_ = 13.951, *p* = .002), but there was no effect of treatment or initial sociability on MMR (Table S3; Figure 5b). There was a significant interacting effect of treatment × mass on AS (*F*_1, 13_ = 6.645, *p* = .023; Table S3). AS was positively correlated with body mass in individuals from both sociable and unsociable groups, but this correlation was stronger in the sociable groups (unsociable: *r* = .133, *p* = .733; sociable: *r* = .930, *p* < .001; Figure 5b).

The LMM analyses showed that swim performance (*U*_crit_) and metabolic rates (SMR, MMR, and AS) of individuals were not related to their current sociability, which was measured after 2 months of experimental group‐living (Table S4).

## 4 DISCUSSION

In this experimental study, the unsociable groups, made up of a high proportion of relatively unsociable juvenile sticklebacks, showed bolder foraging behavior than did the sociable groups. Fish within the groups in which the behavior of the majority matched their own initial sociability (i.e., SS and UU) gained more mass than those within the mismatched groups (i.e., SU and US). The expected positive relationship between body mass and SMR (Clarke & Johnston, 1999) was obscured in the experimental fish mainly because the relatively unsociable individuals within the sociable groups had relatively low mass but had a high SMR. Individual sociability changed during two months of the experimental group‐living in all four types of the experimental fish (SS, SU, UU, and US), but the level of changed sociability was not related to their metabolic rate measured after group‐living. Together, our results suggest that the mismatch between individual and group behavior patterns confers a growth disadvantage, possibly because of limited flexibility in metabolic rate in response to the social environment, which influences the individual's energy turnover. However, the effects of the experimental social environment on individual performance and metabolic flexibility are perhaps inconclusive in our results due to the limited statistical power caused by the small sample sizes, both in numbers of groups and individuals.

Our results show that the groups composed of a high proportion of relatively sociable fish were consistently more fearful than the unsociable groups, with more individuals sheltering under cover before feeding and foraging away from the water surface in the presence of the observer. While all experimental groups, both sociable and unsociable, became increasingly bold throughout the repeated observations probably due to habituation or risk assessment, the differences in collective behaviors between the two treatment groups were maintained. The proportion of individuals seen outside the shelter by the end of 5‐min feeding trials was similar between the sociable and unsociable groups, and almost all fish emerged from the shelter in the last observation. This suggests that information about the presence of food was transmitted rapidly to most fish within the groups irrespectively of the group sociability through either olfactory cues or social transmission (Danchin et al., 2004; Trompf & Brown, 2014). Thus, the differences in collective behaviors between the sociable and unsociable groups were probably maintained merely by consistent individual differences in behavior patterns, habituation, or coping styles (Koolhaas et al., 1999).

Despite the consistent differences in collective behaviors between the sociable and unsociable groups, the difference in individual sociability disappeared over the experimental period, with all categories of fish (SS, SU, US, and UU) showing comparable individual sociability scores after living in groups for two months. In this population, the sociability of juvenile fish is repeatable within individuals in a constant environment (Kim & Velando, 2015). In this study, the initial sociability of the experimental fish was tested after a week of acclimation in isolation to reduce any social influence on the measure of innate social behavior, but the final sociability assay was made while they were still living in the experimental groups. It is possible that the impact of social interactions experienced during the experimental group‐living far outweighed innate sociability in the measurement of individual propensity to stay near others (Moss et al., 2015). Social behaviors are often the most flexible among behavioral traits because the behavior of an individual depends on a complex dynamics of interactions with its conspecifics (Montiglio et al., 2013). It is also possible that the individuals that maintained their initial sociability had a higher mortality rate during the group‐living period than those that changed their behavior patterns (Gordon, 1991).

The consistent differences in collective behaviors over time despite the change in individual sociability suggests that fish within the sociable and unsociable groups were indeed exposed to different social environments, which might be promoted by other group behaviors (e.g., exploration and activity) than group sociability itself. We demonstrated that the experimental social environment had a strong impact on growth of juvenile sticklebacks as a function of their original sociability. Personality‐related behavioral differences are often linked to specific life‐history strategies (Biro & Stamps, 2008; Réale et al., 2010; Sih et al., 2015), and indeed, there is increasing evidence that proactivity (i.e., high boldness, aggressiveness and activity, and low sociability) is positively related to food intake rate and growth rate (Blight et al., 2015; Brodin & Johansson, 2004; Wilson et al., 2013). In this study, the overall rate of mass gain was similar between initially sociable and unsociable fish, but a mismatch between individual and group behavior gave rise to an important growth disadvantage.

Relatively sociable fish grew better in the sociable environment than in the unsociable environment. Since sociable sticklebacks show higher conformity to the group than unsociable individuals, those living within the unsociable proactive groups might move faster and more actively than expected from their states (Jolles et al., 2018). The increased activity could confer a growth disadvantage for the sociable fish within the unsociable groups by increasing their energy expenditure. It is also possible that their unsociable proactive mates, which were predominant in the groups, limited their access to food through aggressive interactions and competition (Bergmüller & Taborsky, 2010; Cutts et al., 1998; Montiglio et al., 2013), although food was provided sufficiently and always some food remained until the following day in all experimental groups.

Similarly, relatively unsociable fish also had a growth disadvantage when living in a social environment that mismatched their individual sociability. Since our experimental fish within the sociable groups were generally fearful, hiding under cover and feeding passively, the initially unsociable fish within the sociable groups probably behaved less proactively than expected from their states for behavioral matching. Indeed, a recent study demonstrated that individual zebrafish (*Danio rerio*) observe then match the fearful antipredator behavior of others in the group through fear contagion and stress transmission (Silva et al., 2019). Since proactive individuals tend to have relatively high metabolic rates (Careau et al., 2008; Šíchová et al., 2014), the low levels of exploration and foraging of the sociable groups probably could not support high energy requirements of the initially unsociable individuals and caused poor mass growth. Indeed, the unsociable individuals within the sociable groups had a relatively high SMR for their mass.

Our results suggest that the experimentally arranged mismatch between individual and group behavior disrupted positive feedbacks between state and behavior, which play an important role in producing consistent among‐individual covariance between behavior and state‐related traits (Dall et al., 2004; Sih et al., 2015). The state of an individual includes not only its own features involving individual morphology, physiology, and life history but also those of its social environment, such as the behavior of its social partners (Sih et al., 2015). The US and SU fish perhaps changed their behavior patterns to match their social environment but at the cost of growth.

The growth disadvantage experienced by the US and SU fish suggests that the experimental fish could not successfully cope with the social challenges, and this is possibly due to their limited flexibility in energy metabolism (Auer et al., 2015). In this study, there was no evidence that the SMR, MMR, and AS of sticklebacks changed in response to group sociability. Interestingly, the US fish showed relatively strong swimming ability in comparison to all other categories of fish probably because proactive fish and slim (but not small) fish are expected to swim faster than reactive fish and heavy fish (Brown et al., 2006; Kern et al., 2016). Juvenile sticklebacks perhaps have a limited ability to change their internal states related to energy metabolism in response to their social environment, and thus, the mismatch between individual and group behavior has negative consequences for growth (Norin & Metcalfe, 2019). However, since metabolic rate and swimming performance were measured only after (and not before) the experimental treatment in a limited number of samples in this study, we cannot draw a strong conclusion about the role of flexibility in metabolic rate in the behavioral adaptation to the social environment and its consequences. It is also important to note that, due to small sample sizes, our data probably could not detect subtle interacting effects (if any) of the social environment and individual behavior on the flexibility in metabolic rate and swimming performance.

This study shows how an individual sociability interacts with its social environment and provides evidence that the expression of nonadaptive behaviors to individual state can incur a growth cost. The intrinsic flexibility of energy metabolism may play an important role in behavioral adaptation to the social environment, although we cannot provide strong evidence due to small sample sizes. It is interesting to note that some animals can rationally choose their social groups (Krause & Ruxton, 2002; Reding & Cummings, 2019). Therefore, individuals that differ in sociability may increase their fitness by choosing between different social groups to match their state to the group behaviors.

## CONFLICT OF INTEREST

The authors declare no competing interests.

## AUTHOR CONTRIBUTIONS

**Sin‐Yeon Kim:** Conceptualization (lead); formal analysis (lead); funding acquisition (lead); investigation (equal); project administration (lead); writing – original draft (lead). **Náyade Álvarez‐Quintero:** Funding acquisition (supporting); investigation (equal); writing – original draft (supporting). **Neil Metcalfe:** Conceptualization (supporting); formal analysis (supporting); funding acquisition (supporting); writing – original draft (supporting).

## Supporting information

Supplementary Material

## Data Availability

Data are available in the Figshare digital repository: https://doi.org/10.6084/m9.figshare.14899038.v1.
