# Supplementary material for: Does the match between individual and group behavior matter in shoaling sticklebacks?
Source: Ecol Evol. 2022 Feb 14;12(2):e8581. doi: 10.1002/ece3.8581 (PMC8844133; doi:10.1002/ece3.8581)
Supplement: Supplementary file 1 — Supplementary Material [file ECE3-12-e8581-s001.pdf]

Supplementary figure and tables

Does the match between individual and group behavior matter in shoaling sticklebacks?

Sin-Yeon Kim[1], Náyade Álvarez-Quintero[1] & Neil B. Metcalfe[2]

[1] Grupo Ecoloxía Animal, Torre CACTI, Centro de Investigación Mariña, Universidade de Vigo, 36310 Vigo, Spain

[2] Institute of Biodiversity, Animal Health and Comparative Medicine, University of Glasgow, G12 8QQ Glasgow, UK

Correspondence: Sin-Yeon Kim. E-mail: yeonkim@uvigo.es

Figure S1 Photograph of the experimental group tank.

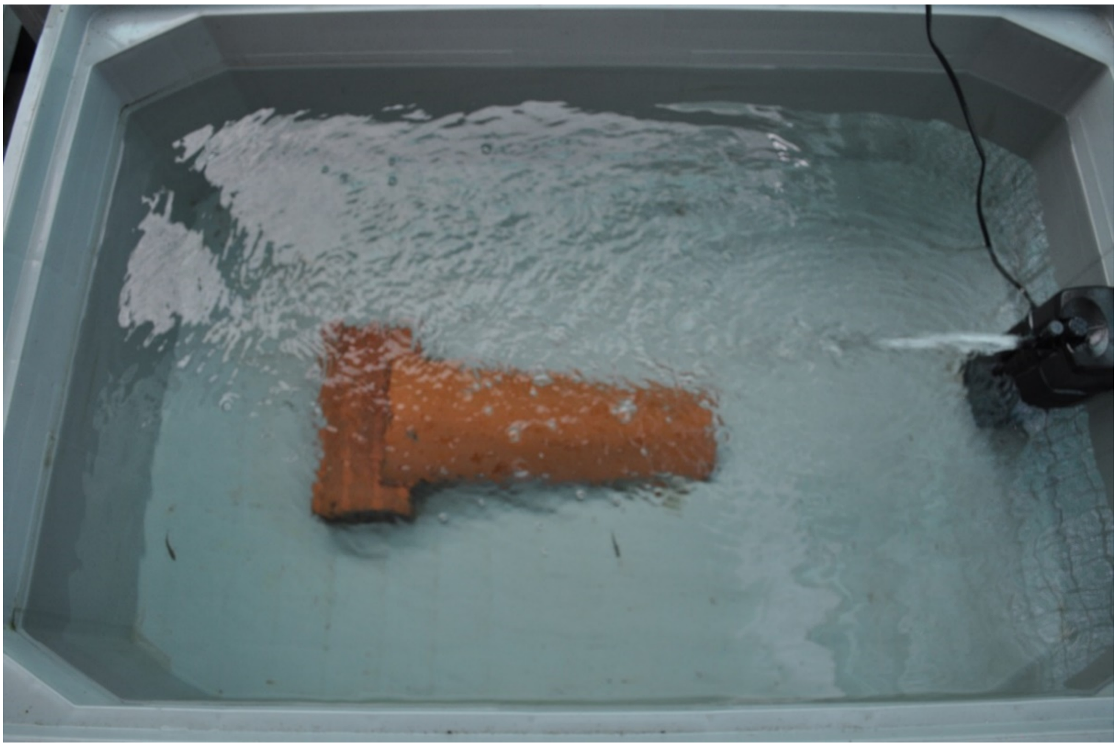

Table S1 Results of generalized linear mixed models (GLMMs) of group behaviors during feeding, exploring the effects of treatment, trial and their interaction. The interaction term was excluded from the final models if statistically not significant.

**Response**	Final model		LRT test	
	Fixed effects	Estimate ± SE	χ^2_1	*p*
**Proportion fish outside the shelter before feeding**				
	Intercept	-0.761 ± 0.322		
	Treatment (sociable)	-0.662 ± 0.264	6.079	**0.014**
	Trial	0.263 ± 0.078	7.075	**0.008**
**Proportion fish outside the shelter after feeding**				
	Intercept	-0.709 ± 0.516		
	Treatment (sociable)	-0.278 ± 0.236	1.383	0.240
	Trial	0.620 ± 0.148	9.942	**0.002**
**Proportion of surface-feeding fish**				
	Intercept	-1.806 ± 0.402		
	Treatment (sociable)	-1.140 ± 0.323	11.875	< **0.001**
	Trial	0.434 ± 0.097	10.656	**0.001**

Table S2 Results of linear mixed models (LMMs) of changes in body mass and length during the experimental group-living. The effects of treatment, initial sociability category, initial mass or size, and treatment × initial sociability were explored; the interaction term was excluded from the final models if not significant.

**Response**	Final model		Type III analysis of variance		
	Fixed effects	Estimate ± SE	*F*	*df*	*p*
**Change in body mass**					
	Intercept	0.171 ± 0.050			
	Treatment (sociable)	-0.057 ± 0.037	0.256	1, 30.98	0.617
	Initial sociability (sociable)	-0.082 ± 0.033	0.262	1, 31	0.613
	Initial body mass	-0.274 ± 0.146	3.522	1, 31	0.070
	Treatment × initial sociability	0.138 ± 0.049	7.875	1, 31	**0.009**
**Change in standard length**					
	Intercept	3.014 ± 2.537			
	Treatment (sociable)	0.007 ± 0.424	< 0.001	1, 32	0.988
	Initial sociability (sociable)	0.059 ± 0.433	0.019	1, 32	0.892
	Initial length	-0.059 ± 0.087	0.457	1, 32	0.504

Table S3 Results of LMMs of U_{crit}, SMR, MMR and AS. The effects of treatment, initial sociability category, body length (U_{crit}) or mass (SMR, MMR and AS), and their two-way and three-way interactions were explored. Interaction terms were excluded from the final models if not significant.

**Response**	Final model		Type III analysis of variance		
	Fixed effects	Estimate ± SE	*F*	*df*	*p*
**U_{crit}**					
	Intercept	7.993 ± 6.847			
	Treatment (sociable)	3.022 ± 2.565	0.077	1, 7.06	0.789
	Initial sociability (sociable)	0.335 ± 1.248	5.319	1, 6.82	0.055
	Standard length	0.058 ± 0.207	0.078	1, 7.77	0.788
	Treatment × initial sociability	-4.720 ± 1.914	6.081	1, 7.08	**0.043**
**SMR**					
	Intercept	0.206 ± 0.065			
	Treatment (sociable)	0.186 ± 0.062	8.984	1, 10.3	**0.013**
	Initial sociability (sociable)	-0.198 ± 0.073	7.301	1, 11.8	**0.019**
	Body mass	-0.108 ± 0.142	1.871	1, 8.7	0.206
	Treatment × mass	-0.487 ± 0.149	10.697	1, 9.3	**0.009**
	Initial sociability × mass	0.506 ± 0.179	7.947	1, 11.7	**0.016**
**MMR**					
	Intercept	0.140 ± 0.095			
	Treatment (sociable)	0.028 ± 0.052	0.294	1, 14	0.596
	Initial sociability (sociable)	0.033 ± 0.052	0.414	1, 14	0.530
	Body mass	0.769 ± 0.206	13.951	1, 14	**0.002**
**AS**					
	Intercept	0.287 ± 0.154			
	Treatment (sociable)	-0.478 ± 0.198	5.865	1, 13	**0.031**
	Initial sociability (sociable)	-0.013 ± 0.057	0.054	1, 13	0.820
	Body mass	0.107 ± 0.330	11.561	1, 13	**0.005**
	Treatment × mass	1.174 ± 0.455	6.645	1, 13	**0.023**

Table S4 Results of LMMs of U_{crit}, SMR, MMR and AS, exploring their relationships with individual sociability score after the experimental group-living.

**Response**	Final model		Type III analysis of variance		
	Fixed effects	Estimate ± SE	*F*	*df*	*p*
**U_{crit}**					
	Intercept	10.672 ± 11.922			
	Final sociability	0.014 ± 0.032	0.206	1, 11.49	0.659
	Standard length	-0.061 ± 0.317	0.038	1, 11.72	0.850
**SMR**					
	Intercept	0.083 ± 0.081			
	Final sociability	0.000 ± 0.000	1.514	1, 14.45	0.238
	Body mass	0.052 ± 0.100	0.265	1, 12.29	0.616
**MMR**					
	Intercept	0.231 ± 0.200			
	Final sociability	-0.000 ± 0.001	0.138	1, 15	0.715
	Body mass	0.725 ± 0.258	7.900	1, 15	**0.013**
**AS**					
	Intercept	0.122 ± 0.231			
	Final sociability	-0.001 ± 0.001	0.384	1, 15	0.545
	Body mass	0.691 ± 0.297	5.411	1, 15	**0.034**
